# Soluble Tumor Necrosis Factor Receptor 1 is Associated With Cardiovascular Risk in Persons With Coronary Artery Calcium Score of Zero

**DOI:** 10.20411/pai.v6i2.477

**Published:** 2021-12-03

**Authors:** Tony Dong, Graham Bevan, David A Zidar, Miguel Cainzos Achirica, Khurram Nasir, Imran Rashid, Sanjay Rajagopalan, Sadeer Al-Kindi

**Affiliations:** 1 Department of Medicine, University Hospitals, Cleveland, Ohio; 2 Louis Stokes Cleveland Veterans Affairs Hospital, Cleveland, Ohio; 3 School of Medicine, Case Western Reserve University, Cleveland, Ohio; 4 Harrington Heart and Vascular Institute, University Hospitals, Cleveland, Ohio; 5 Houston Methodist Hospital, Houston, Texas

**Keywords:** soluble tumor necrosis factor receptor, TNF alpha, coronary calcium score, cardiovascular risk stratification, MESA

## Abstract

**Background:**

A coronary artery calcium (CAC) score of zero confers a low but nonzero risk of atherosclerotic cardiovascular events (CVD) in asymptomatic patient populations, and additional risk stratification is needed to guide preventive interventions. Soluble tumor necrosis factor receptors (sTNFR-1 and sTNFR-2) are shed in the context of TNF-alpha signaling and systemic inflammation, which play a role in atherosclerosis and plaque instability. We hypothesized that serum sTNFR-1 concentrations may aid in cardiovascular risk stratification among asymptomatic patients with a CAC score of zero.

**Methods:**

We included all participants with CAC=0 and baseline sTNFR-1 measurements from the prospective cohort Multi-Ethnic Study of Atherosclerosis (MESA). The primary outcome was a composite CVD event (myocardial infarction, stroke, coronary revascularization, cardiovascular death).

**Results:**

The study included 1471 participants (mean age 57.6 years, 64% female), with measured baseline sTNFR-1 ranging from 603 pg/mL to 5544 pg/mL (mean 1294 pg/mL ±378.8 pg/mL). Over a median follow-up of 8.5 years, 37 participants (2.5%) experienced a CVD event. In multivariable analyses adjusted for Framingham Score, doubling of sTNFR-1 was associated with a 3-fold increase in the hazards of CVD (HR 3.0, 95% CI: 1.48-6.09, *P* = 0.002), which remained significant after adjusting for traditional CVD risk factors individually (HR 2.29; 95% CI: 1.04-5.06, *P*=0.04). Doubling of sTNFR-1 was also associated with progression of CAC >100, adjusted for age (OR 2.84, 95% CI: 1.33-6.03, *P*=0.007).

**Conclusions:**

sTNFR-1 concentrations are associated with more CVD events in participants with a CAC score of zero. Utilizing sTNFR-1 measurements may improve cardiovascular risk stratification and guide primary prevention in otherwise low-risk individuals.

## INTRODUCTION

The pathogenesis of coronary artery disease (CAD) is complex and is characterized by abnormal lipid metabolism and chronic inflammation, leading to endothelial dysfunction and subintimal deposition of low-density lipoproteins (LDL) [1]. The importance of pro-inflammatory pathways in accelerating atherosclerosis is extensively demonstrated in experimental studies and translates directly to clinical utility [2]. C-reactive protein (CRP), a downstream inflammatory biomarker, has been linked to increased cardiovascular risk independent of traditional risk factors [3] and is included in some risk scores for cardiovascular risk stratification [4]. Lipid-lowering therapies are considered to impart anti-inflammatory effects, and they were shown to decrease cardiovascular events in patients with residual inflammatory risk [5]. Recently, the concept of causality between inflammation and atherosclerosis has been proposed with the CANTOS trial, which showed that anti-inflammatory therapy with canakinumab resulted in a significant reduction in adverse cardiovascular events [6]. Soluble tumor necrosis factor receptors (sTNFR-1 and sTNFR-2) are shed during TNF-α cytokine signaling. Circulating levels of sTNFR-1 and sTNFR-2 have been shown to increase with TNF-α activity in response to pro-inflammatory stimuli [7]. They are useful markers of TNF-α activity due to their long half-lives in the serum. TNF-α has been linked to increased cardiovascular events in a 5-year period post myocardial infarction [8] as well as CAD complexity [9]. Several TNF-alpha blockers are approved for use in patients with chronic inflammatory conditions, such as rheumatoid arthritis and inflammatory bowel disease.

Coronary artery calcium (CAC) scoring has become increasingly utilized for cardiovascular risk stratification and is endorsed by the guidelines in intermediate-risk patients. The degree of vascular intimal calcification is directly related to progression of atherosclerosis, and extensive evidence has shown that CAC of zero is associated with very low risk of CVD risk in asymptomatic populations [10–12]. However, a CAC score does not account for noncalcified coronary plaques, which are often less stable and more prone to plaque rupture [13]. Noncalcified coronary plaques are more prevalent in patients with inflammatory diseases (eg, rheumatoid arthritis, HIV, lupus)[14], suggesting a possible correlation with increased systemic inflammation. Thus, it is possible that inflammatory biomarkers increase CVD risk even in patients with zero coronary artery calcium. Here, we investigated the role of serum sTNFR-1 as an inflammatory biomarker in cardiovascular risk stratification for asymptomatic persons with a CAC score of zero.

## METHODS

### Population

Data was obtained from the Multi-Ethnic Study of Atherosclerosis (MESA). The MESA is a prospective cohort study that enrolled 6814 asymptomatic adult participants aged 45 to 84 years between July 2000 and August 2002 from 6 centers in the United States [15]. Baseline characteristics were obtained including demographics, physical exams, cardiovascular risk factors, and CAC score [16]. The CAC scores were obtained using electron-beam and multi-detector row CT. All patients were scanned twice at the first visit and at 5 subsequent follow-up visits, and the mean CAC Agatston score for each visit was used for our analysis. As part of the MESA Family ancillary study, serum sTNFR-1 concentrations were also measured at baseline in a subgroup of 2871 race/ethnicity matched patients. Serum sTNFR-1 concentrations were measured using ultrasensitive ELISA (R&D Systems, Minneapolis, Minnesota) at a central laboratory (University of Vermont, Burlington, Vermont). The MESA Family ancillary study selected an ethnically diverse population for genetic studies and included an even number of patients from each ethnicity (White, African American, Asian, and Hispanic), resulting in a larger proportion of Asians (23.7%) compared to the original MESA cohort (11.6%). We also explored additional inflammatory markers (C-reactive protein [CRP] and Interleukin-6 [IL-6]) for comparative purposes. Serum CRP levels were measured using the BNII nephelometer (N High Sensitivity CRP, Dade Behring, Deerfield, IL; CV 2.1% −5.7%); the lower limit of detection was 0.16 mg/mL. IL-6 was measured by ultra-sensitive sandwich ELISA (Quantikine HS Human IL-6 Immunoassay; R&D Systems, Minneapolis, Minnesota; CV 6.3%). For our study, we included all participants with a CAC score of zero and with measured baseline serum sTNFR-1 concentrations at initial study enrollment. A total of 1471 patients met these criteria. As a part of the MESA study, these participants were followed up over a 10-year period for all CVD events. CVD events were defined as a composite of myocardial infarction (MI), resuscitated cardiac arrest, definite angina, probable angina if followed by revascularization, stroke, and CVD death. Hard CVD events were defined as a composite of myocardial infarction, resuscitated cardiac arrest, cardiovascular disease-related death, stroke, or stroke-related death. All events were adjudicated by a central committee as previously described [17].

### Statistical Analysis

All patients were initially stratified into baseline CAC = 0 and CAC > 0, and mean sTNFR-1 measurements were compared using analysis of variance (ANOVA). Then, baseline characteristics for patients with CAC=0 were calculated including demographics (age, sex, and race), baseline lipid panel (low density lipoprotein (LDL), high density lipoprotein (HDL), triglycerides (TG) and total cholesterol (TC)), and traditional cardiovascular risk factors (family history of MI, history of hypertension, obesity, diabetes, and smoking). Race was divided into categories of White, African American, Asian, and Hispanic. Obesity was divided into body mass index (BMI) categories of <25, 25-30, 30-40, and >40 kg/m^2^. Diabetes was divided into categories of no diabetes, pre-diabetes (defined as fasting glucose ≥ 100 mg/dL), untreated diabetes, and treated diabetes based on a fasting glucose cutoff ≥ 126 mg/dL. Smoking history was a binary variable (current smoker and non-current smoker). Patients were then divided into sTNFR-1 tertiles, and baseline characteristics were calculated for each subgroup. Baseline characteristics were compared between each subgroup using ANOVA for continuous variables and chi-square analysis for categorical variables.

Kaplan-Meier curves were constructed for all participants using time to all CVD events, with censoring of participants who left the study prior to completion. Kaplan-Meier curves were also constructed for each sTNFR-1 tertile subgroup. To measure the association between sTNFR-1 and all CVD events, a multivariate Cox proportional-hazards regression model was implemented using the binary logarithm of sTNFR-1 (log_2_sTNFR-1) as a continuous variable and including traditional cardiovascular risk factors (age, sex, race, diabetes, smoking, total cholesterol, HDL cholesterol, systolic blood pressure, diastolic blood pressure, and antihypertensive medication use). This was repeated for hard CVD events (composite of MI, death from MI, resuscitated cardiac arrest, stroke, and death from stroke), MI alone, stroke alone, and deaths alone. Subgroup analysis was performed on participants stratified by age (≥55 years, <55 years), sex, and Risk Score (≥7.5, <7.5). The predicted 10-year risk of all CVD events with respect to serum sTNFR-1 concentration was calculated using a Cox proportional-hazards regression model adjusting for Framingham Risk Score. The C-statistic was calculated using the area under the curve (AUC) of the receiver operating characteristics (ROC) for the Framingham Risk Score alone, and the Cox proportional-hazards regression model for Framingham Risk Score and sTNFR-1. ROCs were also calculated for 2 other inflammatory markers: interleukin-6 and CRP. The DeLong test was used to compare AUCs. All survival analyses were performed in R version 4.0.4 using the packages survival and rms. All AUC calculations were performed using the package pROC. The distribution of calcium score progression in 4 subsequent visits, stratified by sTNFR-1 tertile was compared using chi-square analysis.

## RESULTS

A total of 6814 participants were included in the MESA. There were 3390 participants (49.8%) who had a baseline CAC of zero, and 1471 participants (mean age 57.6 ±9.1, 35.9% men) had available sTNFR-1 measurements and were included in our study. sTNFR-1 measurements ranged from 603 pg/mL to 5544 pg/mL (mean 1294 pg/mL ±378.8 pg/mL), which was lower than participants with a baseline CAC > 0 (n=1400, mean 1466 pg/mL ± 468.5 pg/mL, *P*<0.001). The baseline characteristics at initial enrollment are reported for the entire population and by sTNFR-1 tertiles (Table 1). sTNFR-1 was associated with older age, male sex, White race, obesity, diabetes, and smoking.

**Table 1.** Participant characteristics by sTNFR-1 tertiles

Participant Characteristics	All	sTNFR-1 tertile 1* (N=489)	sTNFR-1 tertile 2* (N=490)	sTNFR-1 tertile 3* (N=492)	*P* value
Age (years, SD)	57.6 (9.1)	54.8 (7.6)	57.1 (8.5)	61.0 (10.0)	<0.001
Sex					
Male	528 (35.9%)	149 (30.3%)	191 (39%)	188 (38.4%)	0.006
Female	943 (64.1%)	343 (69.7%)	299 (61%)	301 (61.6%)	
Race					
White	322 (21.9%)	90 (18.3%)	110 (22.4%)	122 (24.9%)	
African American	407 (27.7%)	146 (29.7%)	137 (28%)	124 (25.4%)	<0.001
Asian	349 (23.7%)	155 (31.5%)	110 (22.4%)	84 (17.2%)	
Hispanic	393 (26.7%)	101 (20.5%)	133 (27.1%)	159 (32.5%)	
Hypertension	958 (65.1%)	359 (73%)	334 (68.2%)	265 (54.2%)	<0.001
Obesity					
BMI < 25 kg/m^2^	475 (32.3%)	221 (44.9%)	155 (31.6%)	99 (20.2%)	
BMI 25 – 30 kg/m^2^	560 (38.1%)	182 (37%)	184 (37.6%)	194 (39.7%)	<0.001
BMI 30 – 40 kg/m^2^	380 (25.8%)	83 (16.9%)	138 (28.2%)	159 (32.5%)	
BMI > 40 kg/m^2^	56 (3.8%)	6 (1.2%)	13 (2.7%)	37 (7.6%)	
Diabetes mellitus, type II					
None	1139 (77.6%)	400 (81.5%)	397 (81%)	342 (70.2%)	
Insulin Resistance	195 (13.3%)	53 (10.8%)	60 (12.2%)	82 (16.8%)	<0.001
Diabetes, untreated	31 (2.1%)	15 (3.1%)	7 (1.4%)	9 (1.8%)	
Diabetes, on treatment	103 (7%)	23 (4.7%)	26 (5.3%)	54 (11.1%)	
Current Smoker	201 (13.7%)	52 (10.6%)	73 (14.9%)	76 (15.5%)	0.048
Family history of MI	469 (33.8%)	126 (27%)	161 (34.8%)	182 (39.9%)	<0.001
Lipid Profile (mg/dL, SD)					
LDL-C (mg/dL)^1^	115.5 (30.0)	114.3 (29.9)	119.6 (29.7)	112.6 (29.8)	0.381
HDL-C (mg/dL)^2^	52.1 (14.5)	54.9 (15.5)	51.7 (13.9)	49.65 (13.7)	<0.001
TG (mg/dL)^3^	124.6 (65.2)	116.9 (62.4)	122.2 (62.0)	134.8 (69.8)	<0.001
TC (mg/dL)^4^	192.5 (33.0)	192.6 (32.3)	195.7 (32.5)	189.2 (33.9)	0.111

* Tertile 1: 603 – 1118 pg/mL, Tertile 2: 1119 – 1367 pg/mL, Tertile 3: 1368 – 5544 pg/mL

1 Low-density lipoprotein cholesterol

2 High-density lipoprotein cholesterol

3 Triglycerides

4 Total Cholesterol

**Figure 1.**
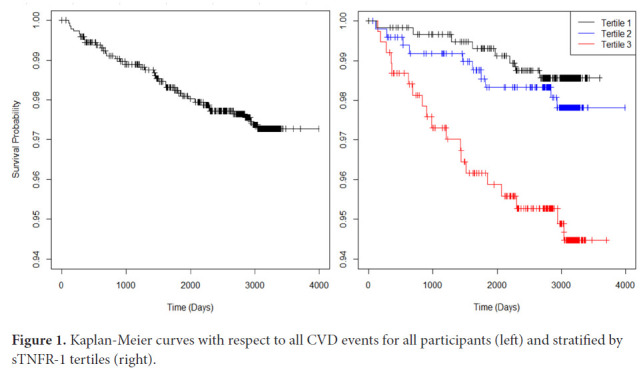
Kaplan-Meier curves with respect to all CVD events for all participants (left) and stratified by sTNFR-1 tertiles (right).

Over the total follow-up period of 10.9 years, 37 participants (2.5%) experienced a CVD event, of which 31 were hard events (12 MI events, 14 stroke events, 1 stroke death, and 4 other cardiovascular deaths). There were also 46 non-cardiovascular deaths. The Kaplan-Meier curve for all CVD events is shown for the entire population and stratified by sTNFR-1 tertiles (Figure 1).

**Figure 2.**
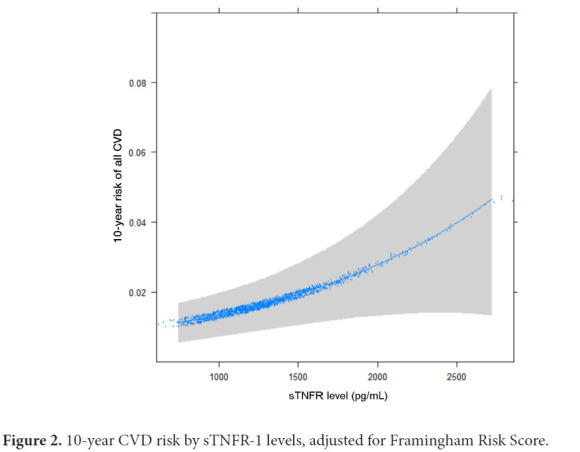
10-year CVD risk by sTNFR-1 levels, adjusted for Framingham Risk Score.

The binary logarithm of sTNFR-1 (log_2_sTNFR-1) was a significant predictor of all CVD events (HR 2.29 [1.04, 5.06], *P=*0.04) after accounting for traditional cardiovascular risk factors. Smoking was also a strong positive predictor (HR 3.65 [1.69, 7.90], *P=*0.001) and African American race was protective compared to White race (HR 0.36 [0.14, 0.92], *P=*0.033). In multivariable analysis adjusted for Framingham Score, doubling of sTNFR-1 was associated with 3-fold increase in risk of CVD (HR 3.0, 95% [1.48, 6.09], *P=*0.002) (Table 2). The relationship of sTNFR-1 levels with modelled 10-year risk of all CVD (adjusted for Framingham Risk Score) is shown (Figure 2). For hard CVD events, log_2_sTNFR-1 continued to be a strong predictor (HR 2.84 [1.223, 6.576], *P=*0.015), along with smoking (HR 3.59 [1.52, 8.45], *P=*0.003).

**Table 2.** Cox proportional-hazards regression models for all CVD for the binary logarithm of sTNFR-1.

	All CVD (N=37)		
	HR	95% CI	P
**Unadjusted log_2_ (sTNFR-1)^1^**	5.15	(2.68, 9.90)	<0.001
**log_2_ (sTNFR-1) adjusted for cardiovascular risk factors^2^**	2.29	(1.04, 5.06)	0.040
**log_2_ (sTNFR-1) adjusted for Framingham Risk Score**	3.00	(1.48, 6.09)	0.002

1 binary logarithm of sTNFR-1

2 Risk factors included: age, sex, race, diabetes, smoking, total cholesterol, HDL cholesterol, systolic blood pressure, diastolic blood pressure, and antihypertensive medication use.

**Figure 3.**
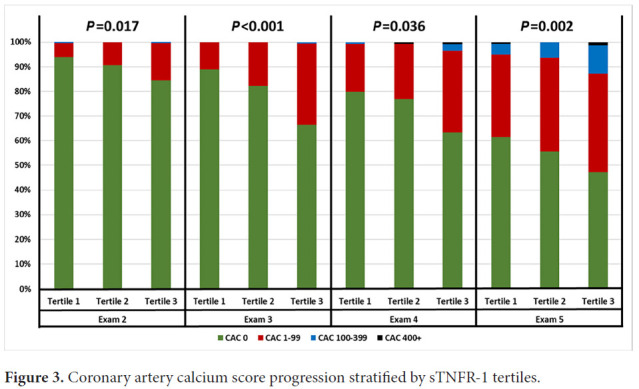
Coronary artery calcium score progression stratified by sTNFR-1 tertiles.

For prediction of all CVD events, the AUC of the ROC of Framingham Risk Score alone was 0.721, compared to an AUC of 0.737 for a composite model of Framingham Risk Score with log_2_sTNFR-1 (*P=*0.009). In addition, sTNFR-1 tertile was associated with CAC progression at subsequent follow-up visits 3 and 5 (*P*<0.001 and *P=*0.002, respectively), as shown (Figure 3). Doubling of sTNFR-1 was associated with progression of CAC>100 at visit 4 (OR 12.93 [2.24, 74.63], *P=*0.004) and visit 5 (OR 2.84 [1.33, 6.03], *P=*0.007), after adjusting for age.

When analyzed separately, log_2_sTNFR-1 was a statistically significant predictor of stroke events (HR 3.59 [1.04, 12.44], *P=*0.044) and of all-cause mortality (HR 3.03 [1.53, 6.01], *P=*0.002), but did not reach statistical significance for MI events (HR 2.72 [0.797, 9.267], *P=*0.11) after accounting for traditional cardiovascular risk factors.

In subgroup analysis by age, log_2_sTNFR-1 was a significant predictor of all CVD in participants under 55 years (HR 10.0 [1.57, 64.45], *P=*0.015) and 55 years or older (HR 2.68 [1.196, 6.021], *P=*0.017). In subgroup analysis by sex and Framingham Risk Score, log_2_sTNFR-1 was a significant predictor in male participants and a high Framingham Risk Score (≥7.5), but not in female participants or a low Framingham Risk Score (<7.5) (Supplemental Table 1). In comparing sTNFR-1 to other available inflammatory markers for prediction of all CVD events, the AUC of the ROC of sTNFR-1 was 0.671, compared to an AUC of 0.570 for interleukin-6 (*P*=0.06) and AUC of 0.503 for CRP (*P*=0.002) (Supplemental Figure 1).

## DISCUSSION

To our knowledge, this is the first study to investigate the association between sTNFR-1 and CV risk in subjects with CAC of 0. We show that sTNFR-1 is associated with CV risk in this low-risk cohort independently of traditional CVD risk factors and improves the 10-year CVD risk stratification of the Framingham Score.

Systemic inflammation has become an increasingly recognized component in the pathogenesis of atherosclerosis and cardiovascular disease. TNF-α plays a key role in the pathogenesis of systemic inflammation [18]. Mechanistically, TNF-α initiates its pro-inflammatory effects by binding to 2 TNF-α receptors TNFR-1 and TNFR-2. These receptors are initially synthesized as membrane-anchored proteins but are subsequently released as soluble receptors that circulate in the blood (sTNFR-1 and sTNFR-2). When cellular TNF-α activity increases in the presence of pro-inflammatory stimuli, the level of sTNFR-1 and sTNFR-2 correspondingly increase in the blood [7]. Due to the long serum half-life of sTNFR-1, it is a measurable and stable marker of ongoing TNF cytokine activity and thus is a useful correlate to systemic inflammation.

Due to the key role of TNF-α in systemic inflammation, it has been a target for immunological therapies in autoimmune conditions like inflammatory bowel disease [19], rheumatoid arthritis [20], and psoriasis [21]. In coronary artery disease, TNF-α is expressed in atherosclerotic plaque [22] and plays a direct role in plaque destabilization [23]. Indeed, sTNFR-1 has been associated with increased arterial stiffening [24], and elevated TNF-α levels have been associated with recurrent cardiovascular events in post-MI patients [8]. Observational studies have also revealed a decreased incidence of MI and stroke in patients receiving anti-TNF therapies for rheumatoid arthritis compared to immunologic naive patients [25]. In those without established pro-inflammatory conditions, adipose tissue may be a driver of TNF-α levels [26], and some intensive diet/exercise interventions are associated with its reduction [27]. Thus, the association between sTNFR-1 with BMI in our study is consistent with the idea that inflammatory risk may also be modified by lifestyle.

Experimental and observational studies have linked TNF signaling with the pathogenesis of atherosclerosis. TNF has been linked to endothelial injury [11], endothelial apoptosis [28], and impairment of endothelial progenitor cells [29], partly through reactive oxygen and nitrogen intermediates [30, 31], resulting in recruitment of inflammatory cells [32] leading to atherosclerosis progression. Small studies have shown that treatment of patients with chronic inflammatory conditions with TNF inhibitors showed short-term improvement in markers of endothelial function [33, 34]. Our observation linking TNF with progression suggests that sTNFR-1 can act as a marker of residual inflammatory risk, even in patients without evidence of overt atherosclerosis, and warrants further studies for selective inhibition of TNF and traditional CV risk reduction strategies (eg, lipid-lowering therapy) in patients with elevated sTNFR-1.

A CAC score of zero is an important negative risk marker that imparts a CVD free “warranty period” of 3 to 7 years in patients without prior history of CVD [35]. In agreement with this, the 2018 ACC/AHA guidelines recommend against initiation of statins in patients with a CAC of zero as long as the 10-year ASCVD risk is less than 20% [36]. The incidence of CVD in our study was low (2.5%) as expected. However, the consideration of systemic inflammation may be important on an individual level even in patients with a CAC of zero as it could lead to undetected noncalcified plaque [14], an increased rate of plaque formation, and risk of plaque rupture. For these patients, traditional risk factors may not fully capture the individualized 10-year CVD risk. This is further supported by the fact that sTNFR-1 improves the discriminative power of Framingham risk in this population as well as the increased rate of calcium score progression with increasing sTNFR-1 tertiles.

Our study highlights sTNFR-1 as an important inflammatory biomarker to consider in patients with a CAC of zero, as it reflects an increased risk of future CVD events. In the MESA cohort, sTNFR-1 as a predictive marker appears to be better than other commonly used inflammatory markers (interleukin-6 and CRP). In association with other traditional risk scores, it may offer added value in CVD risk stratification. It may be reasonable to initiate lipid-lowering therapies or aggressive lifestyle interventions in such patients without CAC, but with a high level of systemic inflammation.

One limitation in this study is the relatively low incidence of CVD due to the low-risk population and relatively short follow-up time, resulting in reduced statistical power. Additionally, this study does not prove causation between sTNFR-1 and CVD events. However, this analysis is strengthened by using a prospective community-based cohort with adjudicated cardiovascular events. Lastly, the upper limit of CVD risk in this patient population did not reach the 7.5% (a common cut-off used for statin initiation). This was likely due to the low-risk profile of this asymptomatic cohort and the relatively low sTNFR-1 values compared to patients with autoimmune conditions like inflammatory bowel disease [37]. Future work should investigate further the utility of adding sTNFR-1 as a biomarker for CVD risk stratification, as well as the benefit of statins in patients with systemic inflammation despite a zero CAC score.

In conclusion, sTNFR-1 concentrations are associated with an increased CVD risk in patients with a CAC of zero, independently of traditional CVD risk factors. Utilizing sTNFR-1 measurements may help to improve cardiovascular risk stratification and guide primary prevention interventions in the future. These findings may have implications for CVD prevention in patients with chronic inflammatory disease and CAC score of zero.

## SUPPLEMENTARY DATA

Supplementary materials are available at the Pathogens and Immunity website. Supplementary data may be provided by the authors to benefit the reader. Supplementary data are not copyedited and are the sole responsibility of the authors. Questions or comments related to supplementary materials should be addressed to the corresponding author.
