# Supplementary material for: Soluble Tumor Necrosis Factor Receptor 1 is Associated With Cardiovascular Risk in Persons With Coronary Artery Calcium Score of Zero
Source: Pathog Immun. 2021 Dec 3;6(2):135–48. doi: 10.20411/pai.v6i2.477 (PMC8714175; doi:10.20411/pai.v6i2.477)
Supplement: Supplementary Tables 1 and 2 [file pai-6-135-s01.pdf]

PATHOGENS AND IMMUNITY
IN ASSOCIATION WITH CASE WESTERN RESERVE UNIVERSITY AND THE RICHARD J. FASENMYER FOUNDATION

Supplementary Table 1. Multivariate Cox proportional-hazards model for all CVD by subgroup (age, sex, and Framingham Risk Score), adjusted for Framingham Risk Score.

**All CVD**		**Hazard Ratio of $\log_2$ sTNFR-1**			
	N	HR	95% CI	P	$P_{interaction}$
**Age ≥ 55 years**	812	2.68	(1.196, 6.021)	**0.017**	0.63
**Age < 55 years**	659	10.0	(1.566, 64.45)	**0.015**	
**Male Sex**	528	3.50	(1.173, 10.447)	**0.025**	0.66
**Female Sex**	943	2.46	(0.968, 6.266)	0.059	
**10-Year Framingham Risk Score < 7.5%**	728	2.51	(0.424, 14.808)	0.311	0.34
**10-Year Framingham Risk Score ≥ 7.5%**	743	2.66	(1.189, 5.939)	**0.017**	

Dong T, Bevan G, Zidar DA, Achirica MC, Nasir K, Rashid I, Rajagopalan S, Al-Kindi S. Soluble Tumor Necrosis Factor Receptor 1 is Associated with Cardiovascular Risk in Persons with Coronary Artery Calcium Score of Zero. *Pathogens and Immunity*. 2021;6(2): 135-148. doi: 10.20411/pai.v6i2.477

PATHOGENS AND IMMUNITY
IN ASSOCIATION WITH CASE WESTERN RESERVE UNIVERSITY AND THE RICHARD J. FASENMYER FOUNDATION

Supplementary Table 2. Baseline characteristics of the subgroup of patients with CAC=0 with measured sTNFR-1 compared with all patients with CAC=0.

**Patient Characteristics**	**All CAC = 0 (N=3390)**	**CAC = 0 with measured sTNFR-1 (N= 1471)**	**CAC = 0 without measured sTNFR-1 (N=1919)**	**p value**
**Age (years, SD)**	57.9 (9.1)	57.6 (9.1)	58.2 (9.2)	0.075
**Sex**				
Male	1238 (36.5%)	528 (35.9%)	710 (37.0%)	0.531
Female	2152 (63.5%)	943 (64.1%)	1209 (63.0%)	
**Race**				
White	1120 (33.0%)	322 (21.9%)	798 (41.6%)	<0.001
African American	1062 (31.3%)	407 (27.7%)	655 (34.1%)	
Asian	393 (11.6%)	349 (23.7%)	44 (2.3%)	
Hispanic	815 (24.0%)	393 (26.7%)	422 (22.0%)	
**Hypertension**	2197 (64.8%)	958 (65.1%)	1239 (64.6%)	0.648
**Obesity**				0.298
BMI < 25	1009 (29.8%)	475 (32.3%)	534 (27.8%)	
BMI 25 - 30	1279 (37.7%)	560 (38.1%)	719 (37.5%)	
BMI 30 - 40	958 (28.3%)	380 (25.8%)	578 (30.1%)	
BMI > 40	144 (4.2%)	56 (3.8%)	88 (4.6%)	
**Diabetes mellitus, type II**				0.159
None	2656 (78.3%)	1139 (77.6%)	1517 (79.1%)	
Insulin Resistance (fasting glucose ≥ 100)	401 (11.8%)	195 (13.3%)	206 (10.7%)	
Diabetes, untreated (fasting glucose ≥ 126)	70 (2.06%)	31 (2.1%)	39 (2.0%)	
Diabetes, on treatment (fasting glucose ≥ 126)	249 (7.35%)	103 (7%)	146 (7.6%)	
**Current Smoker**	449 (13.2%)	201 (13.7%)	248 (12.9%)	0.562
**Family history of MI**	1192 (35.1%)	469 (33.8%)	723 (37.7%)	<0.001
**Lipid Profile (mg/dL, SD)**				
Low density lipoprotein cholesterol (LDL-C)	116.0 (30.9)	115.5 (30.0)	116.5 (31.6)	0.364
High density lipoprotein cholesterol (HDL-C)	52.6 (15.0)	52.1 (14.5)	53.0 (15.3)	0.053
Triglycerides (TG)	121.6 (64.5)	124.6 (65.2)	119.3 (63.8)	0.002
Total cholesterol (TC)	193.0 (34.2)	192.5 (33.0)	193.3 (35.2)	0.878

Dong T, Bevan G, Zidar DA, Achirica MC, Nasir K, Rashid I, Rajagopalan S, Al-Kindi S. Soluble Tumor Necrosis Factor Receptor 1 is Associated with Cardiovascular Risk in Persons with Coronary Artery Calcium Score of Zero. *Pathogens and Immunity*. 2021;6(2): 135-148. doi: 10.20411/pai.v6i2.477
