# Supplementary material for: Soluble Tumor Necrosis Factor Receptor 1 is Associated With Cardiovascular Risk in Persons With Coronary Artery Calcium Score of Zero
Source: Pathog Immun. 2021 Dec 3;6(2):135–48. doi: 10.20411/pai.v6i2.477 (PMC8714175; doi:10.20411/pai.v6i2.477)
Supplement: Supplementary Figure 1 [file pai-6-135-s02.pdf]

PATHOGENS AND IMMUNITY
IN ASSOCIATION WITH CASE WESTERN RESERVE UNIVERSITY AND THE RICHARD J. FASENMYER FOUNDATION

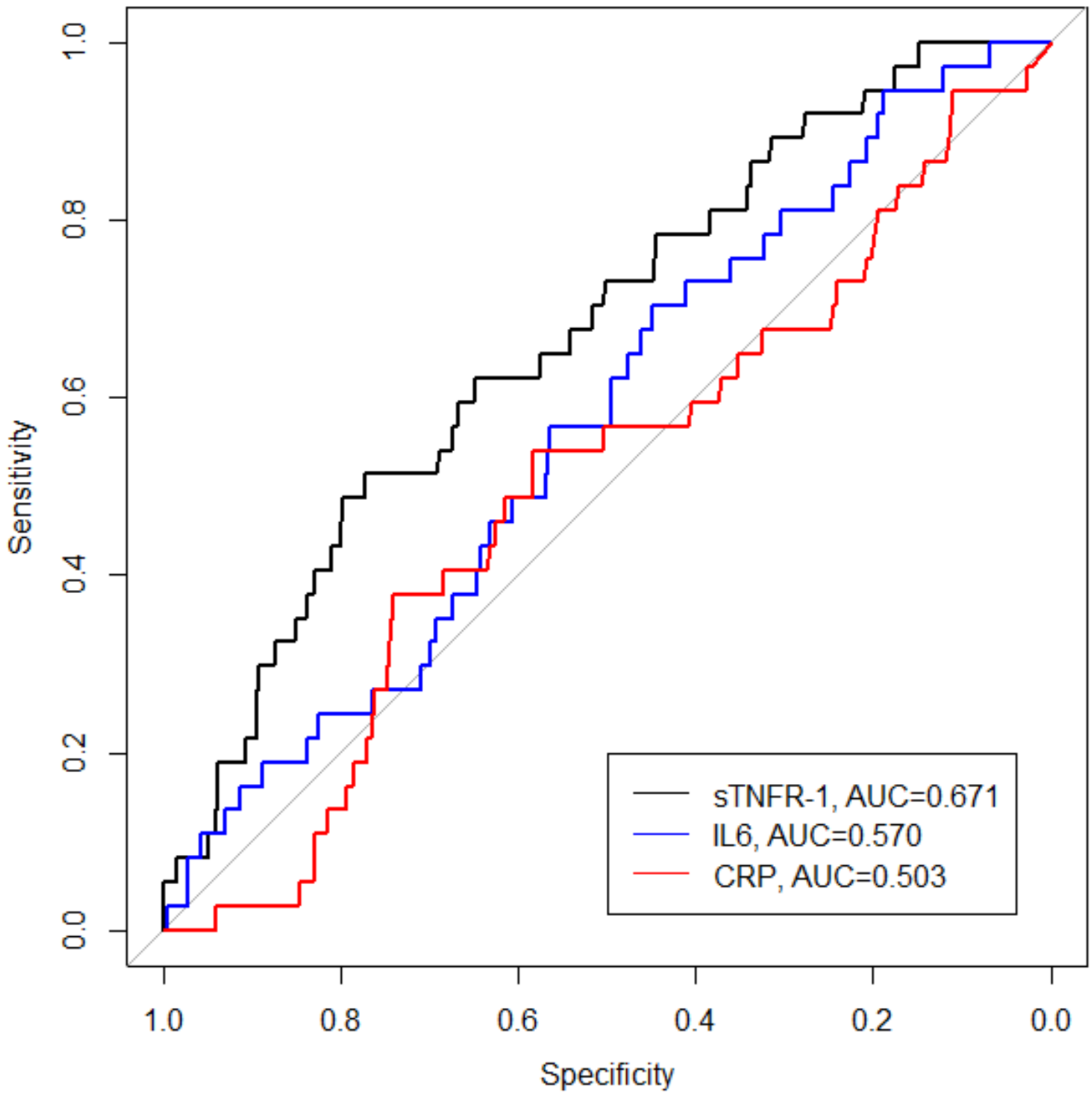

Supplemental Figure 1. Receiver Operator Curve for hard CVD comparing different inflammatory markers: sTNFR-1, interleukin 6 (IL6), and C-reactive peptide (CRP).

Dong T, Bevan G, Zidar DA, Achirica MC, Nasir K, Rashid I, Rajagopalan S, Al-Kindi S. Soluble Tumor Necrosis Factor Receptor 1 is Associated with Cardiovascular Risk in Persons with Coronary Artery Calcium Score of Zero. *Pathogens and Immunity*. 2021;6(2): 135-148. doi: 10.20411/pai.v6i2.477
